# Tackling the Cytotoxic Effect of a Marine Polycyclic Quinone-Type Metabolite: Halenaquinone Induces Molt 4 Cells Apoptosis via Oxidative Stress Combined with the Inhibition of HDAC and Topoisomerase Activities

**DOI:** 10.3390/md13053132

**Published:** 2015-05-20

**Authors:** Shou-Ping Shih, Man-Gang Lee, Mohamed El-Shazly, Yung-Shun Juan, Zhi-Hong Wen, Ying-Chi Du, Jui-Hsin Su, Ping-Jyun Sung, Yu-Cheng Chen, Juan-Cheng Yang, Yang-Chang Wu, Mei-Chin Lu

**Affiliations:** 1 Graduate Institute of Marine Biotechnology, National Dong Hwa University, Pingtung 944, Taiwan; E-Mails: m6430005@hotmail.com (S.-P.S.); x2219@nmmba.gov.tw (J.-H.S.); pjsung@nmmba.gov.tw (P.-J. S.); 2 Department of Marine Biotechnology and Resources, National Sun Yat-sen University, Kaohsiung 804, Taiwan; E-Mails: mg2253@yahoo.com.tw (M.-G.L.); wzh@mail.nsysu.edu.tw (Z.-H.W.); 3 Division of Urology, Department of Surgery, Zuoying Branch of Kaohsiung Armed Forces General Hospital, Kaohsiung 813, Taiwan; E-Mail: mg2253@yahoo.com.tw; 4 Department of Pharmacognosy and Natural Products Chemistry, Faculty of Pharmacy, Ain-Shams University, Organization of African Unity Street, Abassia, Cairo 11566, Egypt; E-Mail: elshazly444@googlemail.com; 5 Department of Urology, Kaohsiung Municipal Hsiao-Kang Hospital, Kaohsiung 812, Taiwan; E-Mail: juanuro@gmail.com; 6 Department of Urology, College of Medicine, Kaohsiung Medical University, Kaohsiung 807, Taiwan; 7 Department of Urology, Kaohsiung Medical University Hospital, Kaohsiung 807, Taiwan; 8 Department of Botanicals, Medical and Pharmaceutical Industry Technology and Development Center , New Taipei City 248, Taiwan; E-Mail: ycdu0626@gmail.com; 9 National Museum of Marine Biology & Aquarium, Pingtung 944, Taiwan; 10 The PhD Program of Cancer Biology and Drug discovery, China Medical University, Taichung 404, Taiwan; E-Mail: j520c@hotmail.com; 11 School of Pharmacy, College of Pharmacy, China Medical University, Taichung 404, Taiwan; E-Mails: q9113054@yahoo.com.tw (J.-C.Y.); yachwu@mail.cmu.edu.tw (Y.-C.W.); 12 Chinese Medicine Research and Development Center, China Medical University Hospital, Taichung 404, Taiwan; 13 Graduate Institute of Natural Products, Kaohsiung Medical University, Kaohsiung 807, Taiwan; 14 Center of Molecular Medicine, China Medical University Hospital, Taichung 404, Taiwan

**Keywords:** halenaquinone, histone deacetylase (HDAC), mitochondria, reactive oxygen species (ROS), topoisomerase

## Abstract

A marine polycyclic quinone-type metabolite, halenaquinone (HQ), was found to inhibit the proliferation of Molt 4, K562, MDA-MB-231 and DLD-1 cancer cell lines, with IC_50_ of 0.48, 0.18, 8.0 and 6.76 μg/mL, respectively. It exhibited the most potent activity against leukemia Molt 4 cells. Accumulating evidence showed that HQ may act as a potent protein kinase inhibitor in cancer therapy. To fully understand the mechanism of HQ, we further explored the precise molecular targets in leukemia Molt 4 cells. We found that the use of HQ increased apoptosis by 26.23%–70.27% and caused disruption of mitochondrial membrane potential (MMP) by 17.15%–53.25% in a dose-dependent manner, as demonstrated by Annexin-V/PI and JC-1 staining assays, respectively. Moreover, our findings indicated that the pretreatment of Molt 4 cells with *N*-acetyl-l-cysteine (NAC), a reactive oxygen species (ROS) scavenger, diminished MMP disruption and apoptosis induced by HQ, suggesting that ROS overproduction plays a crucial rule in the cytotoxic activity of HQ. The results of a cell-free system assay indicated that HQ could act as an HDAC and topoisomerase catalytic inhibitor through the inhibition of pan-HDAC and topoisomerase IIα expression, respectively. On the protein level, the expression of the anti-apoptotic proteins *p*-Akt, NFκB, HDAC and Bcl-2, as well as hexokinase II was inhibited by the use of HQ. On the other hand, the expression of the pro-apoptotic protein Bax, PARP cleavage, caspase activation and cytochrome *c* release were increased after HQ treatment. Taken together, our results suggested that the antileukemic effect of HQ is ROS-mediated mitochondrial apoptosis combined with the inhibitory effect on HDAC and topoisomerase activities.

## 1. Introduction

Protein acetylation is one of the vital post-translational modifications that regulate protein stability, function and intracellular compartmentalization. It is involved in epigenetic regulation through chromatin remodeling. Histone deacetylase inhibitors (HDACi) exert anticancer activity by promoting acetylation of histones, as well as by promoting acetylation of non-histone protein substrates, including α-tubulin, heat shock protein 90 or p53 [1,2]. Accumulating evidence suggested that HDAC activity plays an important role in tumorigenesis (especially leukemia), and thus, these epigenetic modifications could be potential targets for cancer therapy [3,4]. Suberoylanilide hydroxamic acid (SAHA) and romidepsin (depsipeptide) were approved as HDACi for clinical treatment by the U.S. Food and Drug Administration for the treatment of cutaneous T-cell lymphoma in 2006 and 2009, respectively [5,6,7]. There are currently ten HDAC inhibitors in clinical trials, including six hydroxamic acids, two benzamides and two fatty acids [8].

There are several lines of evidence demonstrating that drugs targeting DNA topoisomerase (topo) I and II act as specific DNA-damaging agents and can be used in cancer therapy, because tumor cells could not sustain significant defects in the DNA repair pathway [9]. Topo I poison-targeting drugs used in cancer therapy include camptothecin, irinotecan and topotecan. These agents stabilize the formed cleavable complex via the interfacial inhibition, which induces DNA damage that ultimately leads to cell death. However, *de novo* or acquired clinical resistance to these drugs is common [10]. To overcome such drawbacks, another line of drugs has emerged targeting topo IIα [11]. It was found that topo IIα-mediated ERBB2 co-amplification could lead to the development of tumors accompanied with an increase in the expression of topo IIα, which may be tight relative to the phenotypes of cancer cells [12]. Two types of topo Iiα-targeting agents were developed, including topo IIα poisons and catalytic inhibitors. Clinical drugs acting as topo IIα poisons are epipodophyllotoxin etoposide and anthracycline doxorubicin. They act by increasing the levels of covalent enzyme-cleaved DNA complexes [12]. Unfortunately, these drugs are linked to the development of acute myeloid leukemia through causing rearrangement at chromosomal band 11q23 [13]. On the other hand, it is believed that catalytic inhibitors of topo IIα do not lead to such side effects and could act as potential anticancer drugs.

Xestoquinone and halenaquinone (HQ), which are polycyclic quinone-type metabolites, were found to exhibit various biological activities, such as antifungal, cardiotonic, cytotoxic and topoisomerase activities, as well as acting as inhibitors for different protein kinases [14,15,16,17,18]. In addition, halenaquinone specifically inhibited the secondary DNA binding of RAD51, leading to the accumulation of chromosomal aberrations induced by unrepaired double-strand breaks [19]. Recently, Tsukamoto *et al.* suggested that halenaquinone inhibited RANKL-induced osteoclastogenesis by suppressing the NFκB and Akt signaling pathways [20]. HQ, the marine natural product isolated from the *Petrosia* sponge, is a broad spectrum tyrosine kinase inhibitor and more potent than xestoquinone, with a carbonyl group at the C-3 position. In this study, the cytotoxic and antitumor mechanisms of HQ were further investigated in a human leukemia Molt 4 cellular and xenograft animal model.

## 2. Results

### 2.1. Effect of HQ on Cellular and Tumor Growth in Vitro Assay and in Vivo Animal Model

**Figure 1:**
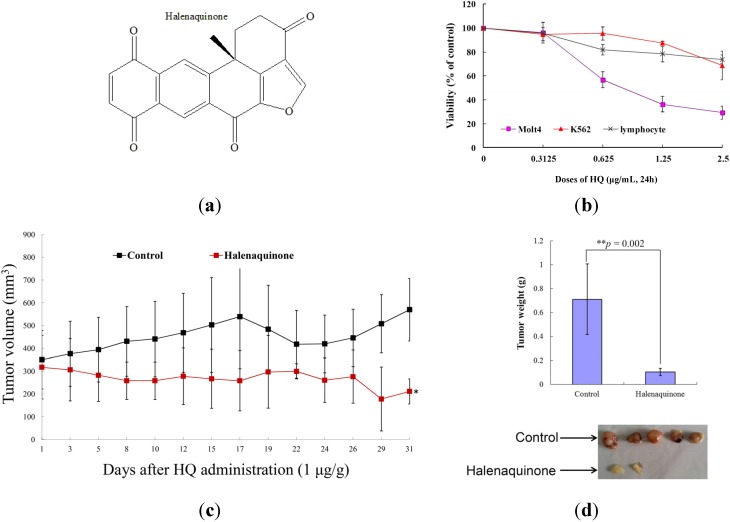
Effect of halenaquinone (HQ) on cellular viability *in vitro* and tumor growth *in vivo* animal model. (**a**) Chemical structure of marine polycyclic quinone-type metabolites, HQ, isolated from *Petrosia* sp. Sponge; (**b**) Human leukemia Molt 4 and K562 cells, as well as normal rat lymphocytes were treated with HQ at different doses for 24 h. The viability was determined by the MTT assay; (**c**) *In vivo* inhibition of tumor growth with human leukemia Molt 4 xenograft by HQ. Female nude mice bearing leukemia Molt 4 tumors were treated with the solvent (negative control, *n* = 8) or HQ (1 μg/g, *n* = 8) for one month. Tumor volumes were measured every other day, and the results are expressed as the mean ± SD. * Significantly different from control groups at *p* = 0.015; (**d**) Histogram of the tumor weight from the control group and HQ-treated group. Values are expressed as the mean ± SD. ** Significantly different from control groups at *p* = 0.002. Representative photos of the subcutaneous tumors, which were collected after treatment with the solvent only (upper) or with HQ (lower) for 31 days.

The cytotoxicity of HQ was evaluated using the MTT assay against various human cancer cell lines. HQ was found to inhibit the proliferation of Molt 4 (human acute lymphoblastic leukemia), K562 (human chronic myelogenous leukemia), MDA-MB-231(human breast adenocarcinoma) and DLD-1 (human colon adenocarcinoma) cancer cells, with IC_50_ of 0.18, 0.48, 8 and 6.76 μg/mL after 72 h, respectively. It exhibited the most potent activity against leukemia Molt 4 and K 562 cells (the values of IC_50_ were less than 4 μg/mL). These findings encouraged us to expand our cytotoxic study aiming to reveal the HQ mechanism of action against leukemia cancer cell lines. To pursue this goal, the cytotoxic effect of HQ against Molt 4 cells was determined after 24 h, resulting in IC_50_ values of 0.61 μg/mL. Furthermore, it was important to determine whether the cytotoxic effect of HQ is specific for cancer cells. The effect of HQ on the viability of rat normal lymphocytes was evaluated. The results indicated that even at the highest dose (2.5 μg/mL), HQ treatment caused only 26.32% suppression in the viability of lymphocytes, but a significant decrease of about 70.78% and 31.33% in Molt 4 and K562 cells (Figure 1b). Thus, it may be concluded that HQ’s cytotoxic effect is more specific towards Molt 4 cells compared to K562 cells.

The *in vivo* anti-tumor activity of HQ was determined by evaluating its effect on the tumor growth of a human leukemia Molt 4 xenograft in an animal model. Molt 4 (1 × 10^5^) cells were inoculated subcutaneously at the right flank of female immunodeficient athymic mice. After one month of treatment, the tumor growth of Molt 4 cells was significantly suppressed under the influence of HQ (1 μg/g) intraperitoneal injection. The average tumor size on Day 31 in the control group was 570.13 mm^3^, whereas the average tumor size in the HQ-treated group was 211.29 mm^3^ (Figure 1c). The tumor size was significantly lower in the HQ-treated group as compared to the control group (*p* = 0.002), with no significant difference in the mice body weights. At the end of the treatment, the tumor tissue was isolated and weighed. The average weights of the tumor were obviously less in the HQ-treated group (0.10 ± 0.04 g) compared to the control group (0.71 ± 0.36 g) (Figure 1d). These results suggested the anti-tumorigenic effect of the HQ *in vivo* xenograft model.

### 2.2. Effect of HQ on Topoisomerase I and II Activity

**Figure 2:**
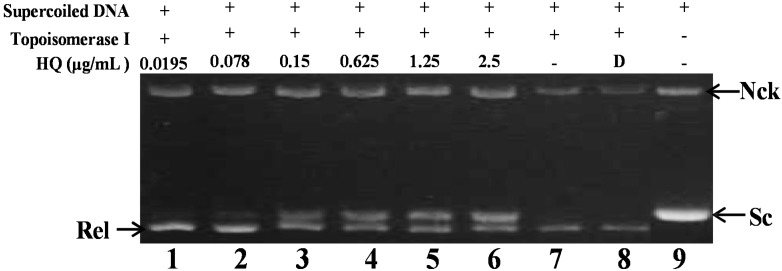
Effect of HQ on topo I activity. Lanes 1–6: HQ (0.0195, 0.078, 0.15, 0.625, 1.25 and 2.5 μg); Lane 7: plasmid DNA + topo I; Lane 8: plasmid DNA + topo I + solvent control (induction of DNA relaxation); Lane 9: negative control plasmid DNA (supercoiled DNA). Nck, nicked DNA; Rel, relaxed DNA; Sc, supercoiled DNA.

As previously described, the marine polyketide analog, xestoquinone, was identified as a topo I inhibitor utilizing calf thymus proteins, which was used to determine the relaxation of ColE1 DNA [21]. To further confirm whether the other polyketide analog, HQ, could inhibit topo I or II activity in the cell-free system, the purified human DNA topoisomerase protein was used to examine the relaxation of supercoiled plasmid DNA [22]. Initially, the effect of HQ on topo I activity was studied. A cell-free DNA cleavage assay using an enzyme-mediated negatively-supercoiled pHOT1 plasmid DNA was applied to study this effect. As shown in Figure 2, HQ at the lowest dose (0.0195 μg) induced DNA relaxation in the presence of topo I (Lane 1); but at higher doses (0.078, 0.15, 0.625, 1.25 and 2.5 μg/mL), it inhibited the ability of topo I to convert supercoiled DNA to the relaxed form (Lanes 2–6). HQ at doses of 0.0195, 0.078, 0.15, 0.625, 1.25 and 2.5 μg/mL significantly decreased the relaxation of supercoiled DNA induced by topo I by 4%, 9%, 30%, 40%, 48% and 64%, respectively, compared to the control supercoiled DNA. As expected, the relaxation of the supercoiled DNA was inhibited by HQ in a dose-dependent manner, with an IC_50_ value of 1.19 μg/mL.

**Figure 3:**
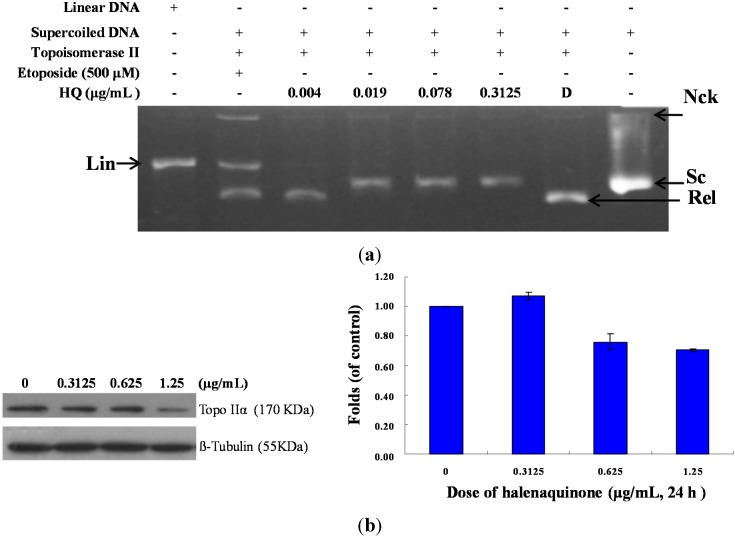
Effect of HQ on topo II activity. (**a**) The effect of HQ on topo Iiα mediated supercoiled pHOT1 plasmid DNA relaxation with the cell-free system. Lane 1: linear DNA; Lane 2: positive control, etoposide (500 μM), as the topo II poison (induction of linear DNA); Lanes 3–6: HQ (0.004, 0.019, 0.078 and 0.3125 μg/mL); Lane 7: plasmid DNA + topo IIα (induction of DNA relaxation); Lane 8: negative control plasmid DNA (supercoiled DNA). Nck, nicked DNA; Rel, relaxed DNA; Sc, supercoiled DNA; Lin, linear DNA; (**b**) HQ decreased the expression of topo IIα protein in Molt 4 cells. Molt 4 cells were treated with HQ (0, 0.3125, 0.625 and 1.25 μg/mL) for 24 h. The protein expression of topo IIα was analyzed with Western blotting. The bands were quantified via densitometry and normalized relative to the β-tubulin levels.

As previously reported, the marine polyketide analogs, xestoquinone and halenaquinone, did not inhibit the activity of calf thymus topo II [21]. Nevertheless, other xestoquinone analogs inhibited topo I and II activity, and they did not intercalate with DNA [23]. In order to confirm HQ’s effect on topo II activity and to understand its actual cytotoxic mechanism of action, a cell-free DNA cleavage assay using an enzyme-mediated negatively-supercoiled pHOT1 plasmid DNA was applied. A linear DNA strand was observed upon treating the supercoiled pHOT1 plasmid DNA with etoposide, a standard topo II poison (Lane 5) [24]. The use of HQ in increasing concentrations (0.004, 0.019, 0.078 and 0.3125 μg/mL) completely inhibited DNA relaxation by 43%, 81%, 90% and 98%, respectively, compared to the control supercoiled DNA and resulted in the formation of supercoiled DNA products in the presence of topo IIα (Lanes 2–6) (Figure 3a). HQ showed comparable activity to adociaquinones A and B [23], resulting in the inhibition of supercoiled DNA relaxation in a dose-dependent manner. In addition, Western blotting indicated that the use of HQ (1.25 μg/mL) significantly diminished topo IIα protein expression (Figure 3b). Furthermore, HQ inhibited topo I and II activities with IC_50_ of 1.19 and 0.0055 μg/mL, respectively as demonstrated by the cell-free system. These results suggested that HQ could act as a dual inhibitor of topo I and II. To further precisely identify the effect of topo I and II, we used the purified human protein, not calf, to examine the inhibitory effect of HQ.

### 2.3. Effect of HQ on Histone Deacetylase Activity

**Figure 4:**
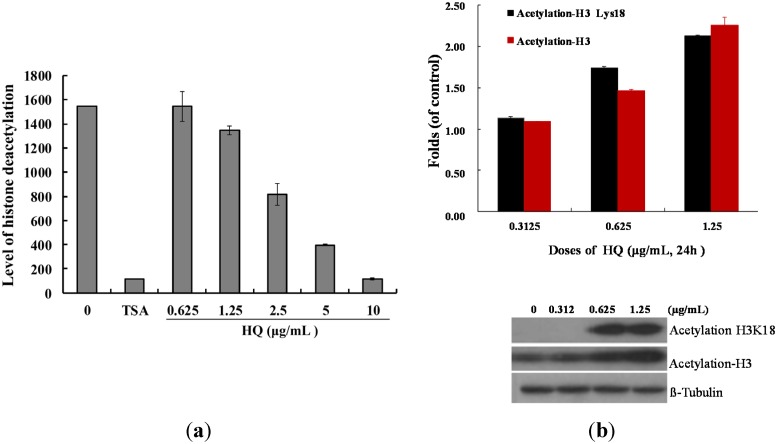
Effect of HQ on histone deacetylase activity. (**a**) The effect of HQ on HDAC mediated deacetylation of lysine side chain with the cell-free system. Trichostatin A (TSA) is a potent reversible inhibitor of HDAC and was used as the positive control. Relative deacetylation of histone was progressively deceased by the treatment of HQ (0.625, 1.25, 2.5, 5 and 10 μg/mL); (**b**) HQ increased the expression of acetyl-H3 and acetyl-H3K18 in Molt 4 cells. Molt 4 cells were treated with HQ (0, 0.3125, 0.625 and 1.25 μg/mL) for 24 h. The protein expression was analyzed with Western blotting. The bands were quantified via densitometry and normalized relative to the β-tubulin levels.

Our results demonstrated a significant inhibitory effect of HQ on the proliferation and tumorigenesis of cancer cells (Figure 1). To evaluate whether HQ can inhibit epigenetic expression, a cell-free HDAC colorimetric acetylated lysine side chain assay using an enzyme-mediated deacetylation was used. As shown in Figure 4a, HQ inhibited deacetylation of HDAC activity compared to the solvent control with an IC_50_ value of 2.95 μg/mL, and the effect was in a concentration-dependent manner. Next, the effect of HQ on the level of histone acetylation of Molt 4 cells was examined with Western blotting analysis. A significant acetylation of H3 was observed at doses of 0.625 and 1.25 μg/mL after 24 h with an increase of 1.14- and 2.28-fold (acetyl-H3), as well as 1.74- and 2.13-fold (acetyl-H3K18) compared to the control group, respectively (Figure 4b).

### 2.4. Effect of HQ on Apoptosis Induction Involved Mitochondrial Dysfunction in Molt 4 Cells

**Figure 5:**
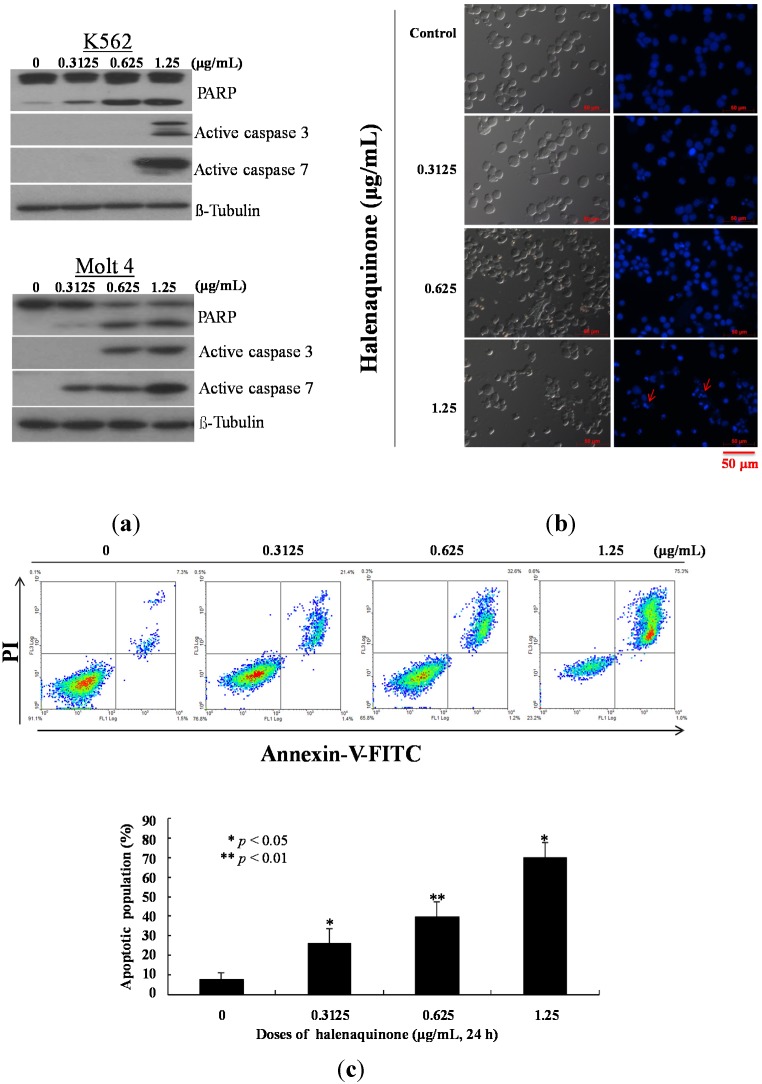

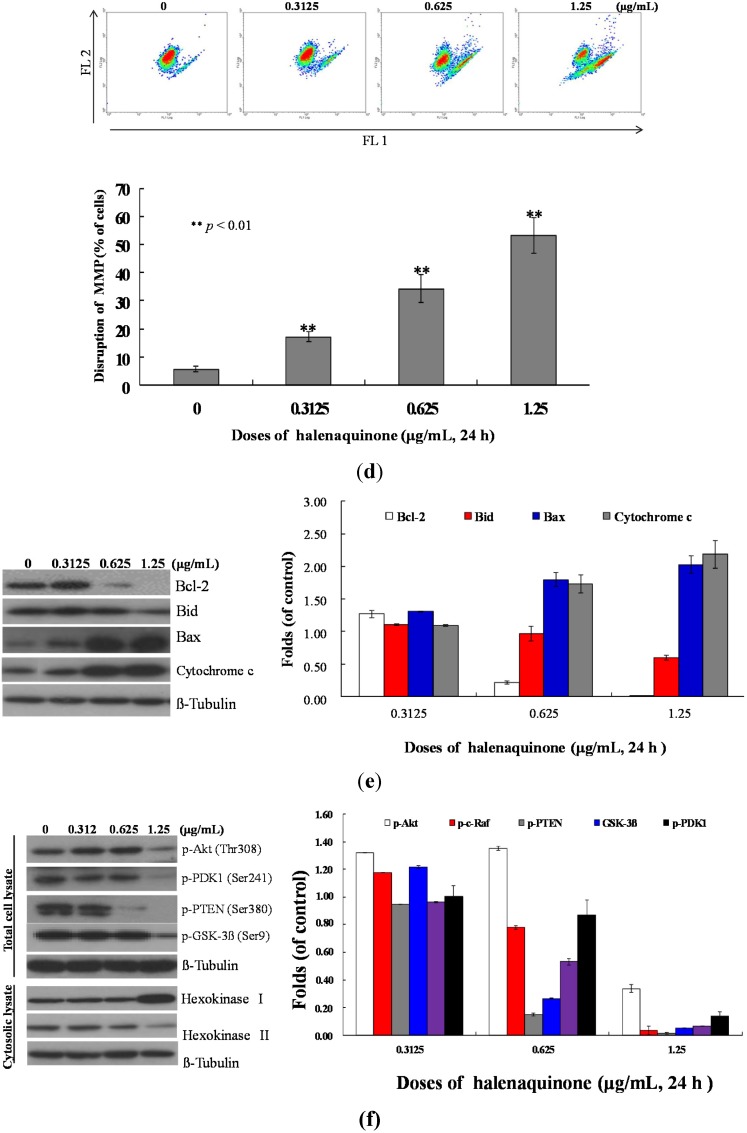

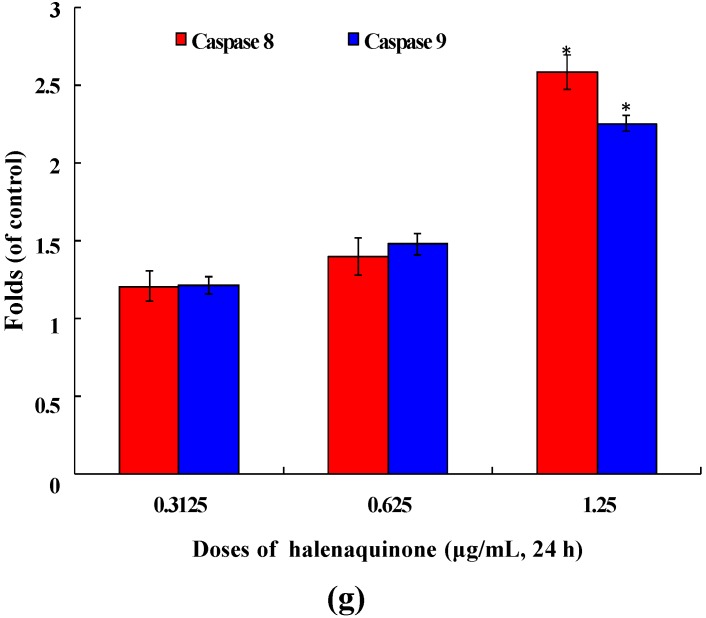
Apoptotic effect of HQ on Molt 4 cells. Cells were treated with the indicated concentration of HQ for 24 h. (**a**) HQ treatment enhanced the cleavage of apoptosis-related proteins in K562 and Molt 4 cells; (**b**) The change of nuclear morphology was determined with DAPI staining and observed using fluorescent microscope; (**c**) Apoptosis induction and (**d**) mitochondrial membrane potential were assessed with annexin V/PI and JC-1 staining using flow cytometric analysis; (**e**) Proapoptosis-and (**f**) Survival-related proteins were determined with Western blotting. The results are presented as means ± SD of three independent experiments (* *p* < 0.05; ** *p* < 0.01); (**g**) After treatment, cytoplasmic lysates (50 μg) were prepared and the enzymatic activity of caspases 8 and 9 like protease was determined by the incubation with colorigenic substrates, for 2 h at 37 °C. The release of chromophore pNA was monitored spectrophotometrically (405 nm).

Previous studies demonstrated a significant cytotoxicity of HQ against several cancer cells [14,17,21]. To examine whether the cytotoxic effect of HQ on K562 and Molt 4 cells involves apoptotic induction, the cells were treated with HQ, and the apoptotic-related proteins (PARP, caspases 3 and 7) were analyzed with Western blotting. As indicated in Figure 5a, the treatment with HQ caused cleavage of PARP at low doses (0.3125 and 0.625 μg/mL) in both types of cells after 24 h. In Molt 4 cells, HQ resulted in an activation of caspases 3 and 7 in a dose-dependent manner. On the other hand, caspases 3 and 7 in K562 cells were activated only after the treatment with 1.25 μg/mL of HQ. To better comprehend the antitumor mechanism of HQ, the apoptotic cells population was determined using Annexin V/PI and DAPI staining assay in Molt 4 cells. After 24 h of treatment, the percentage of apoptotic cell population was significantly increased by 26.23%, 39.93% and 70.27% compared to the solvent control (Figure 5b). To assess the nuclear morphological change induced by HQ, the Molt 4 cells were further examined with DAPI staining and observed under a fluorescence microscope. The results showed that HQ treatment increased the number of condensed nuclei compared to the control, which exhibited intact and normal nuclei (Figure 5c). To address whether the apoptotic induction of HQ was related to the mitochondrial pathway, JC-1 fluorescent dye was used to determine the mitochondrial membrane potential. Molt 4 cells were treated with different doses of HQ for 24 h and then stained with JC-1. As shown in Figure 5d, the use of HQ (0.3125 μg/mL) increased the population of Molt 4 cells with disrupted membrane potential from 6.03% up to 16.36%. This effect was dramatically increased upon the treatment with HQ at 0.625 and 1.25 μg/mL, which resulted in 32.96% and 53.06% cells with disturbed MMP, respectively. To further explore the mechanism of HQ-induced apoptosis, the effect of HQ on apoptotic- and mitochondrial metabolism-related proteins was evaluated.

As shown in Figure 5e, HQ treatment eliminated the expression of the anti-apoptotic proteins Bcl-2 and Bid. Concomitantly, HQ treatment induced the expression of cytochrome *c* and the pro-apoptotic protein, Bax. It increased the expression of cytochrome *c* 1.73- and 2.19-fold, as well as Bax expression 1.8- and 2.03-fold at doses of 0.625 and 1.25 μg/mL, respectively. Moreover, the treatment of leukemia Molt 4 cells with different concentrations of HQ diminished the expression of p-Akt (Ser473), p-PTEN (Ser380), p-GSK3β (Ser9), p-PDK1 (Ser241) and cytosolic hexokinase II, but enhanced the expression of cytosolic hexokinase I about 1.34-fold compared to the control group at the high dose of 1.25 μg/mL (Figure 5f). Caspase-3 is a key executioner of apoptosis, which is activated by an initiator caspase, such as caspases 8 or 9. These activated caspases cleaved many cellular substrates, ultimately leading to cell apoptosis [25]. To explore whether the mechanism of the apoptotic effect of HQ involved the activation of caspase initiator, the cleavage of caspases 8 and 9 was tested using the calorigenic assay. The treatment of Molt 4 cells with the 0.3125, 0.625 and 1.25 μg/mL of HQ led to an increase of the active forms of caspase 8 by about 1.2-, 1.4- and 2.6-fold, as well as the active forms of caspase 9 by about 1.2-, 1.5- and 2.3-fold, respectively (Figure 5g).

### 2.5. HQ Induced Apoptosis Is Mediated by Excessive ROS Generation

**Figure 6:**
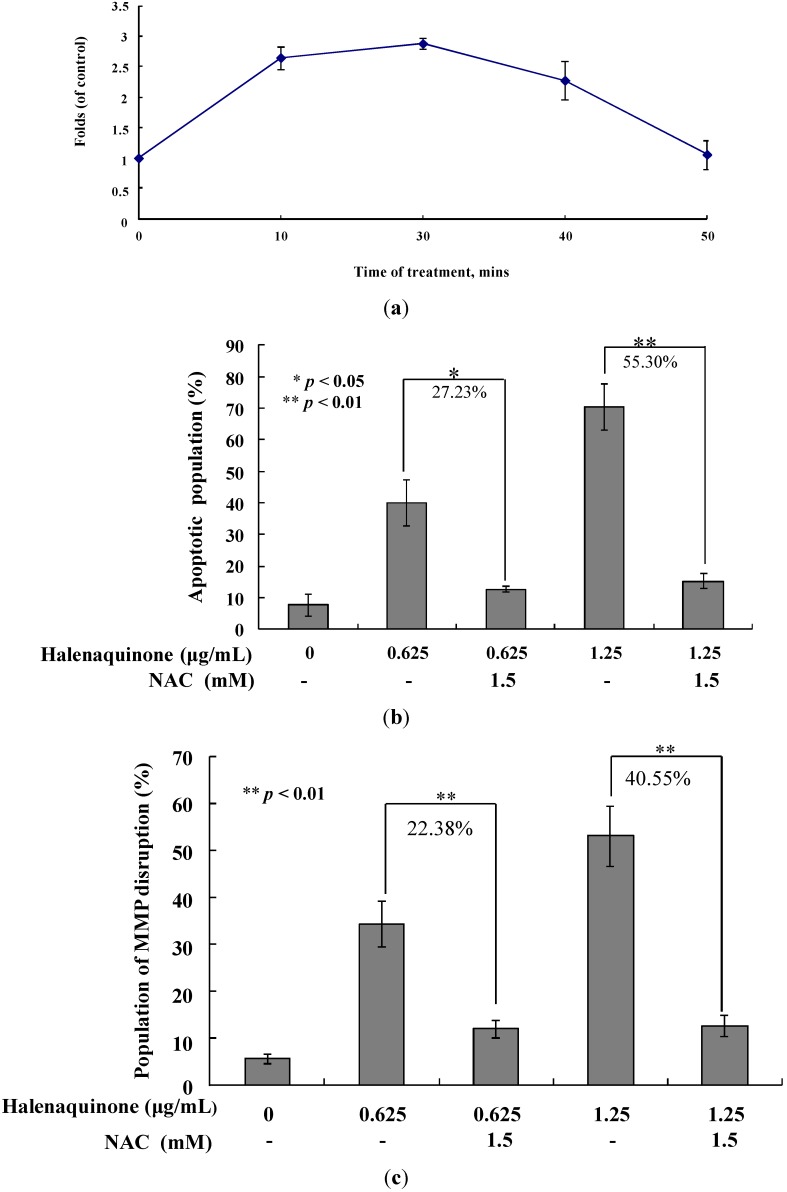

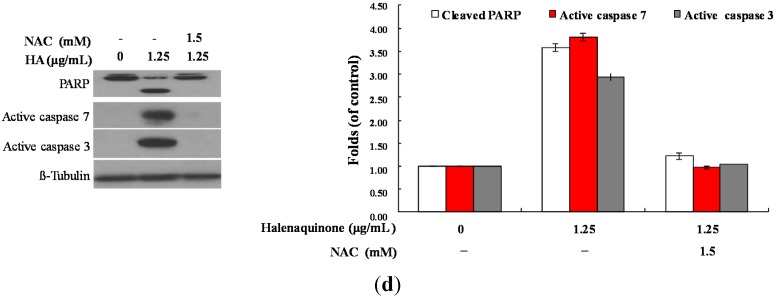
The apoptotic induction of HQ in Molt 4 cells involves ROS production. The effect of HQ treatment on ROS generation in Molt 4 cells was evaluated. (**a**) Cells were treated with HQ (1.25 μg/mL) for the indicated times. Quantitative results showed a gradual increase in the ROS production in response to HQ treatment when compared with the control group. Moreover, the effect of ROS generation on the HQ-induced apoptosis in Molt 4 cells was examined. Cells were pretreated with 1.5 mM NAC for 2 h, then treated with 0.625 or 1.25 μg/mL of HQ; The apoptotic populations (**b**), the disruption of MMP (**c**) and the expression of apoptosis-related protein (**d**) were examined with annexin-V/PI and JC-1 staining as well as Western blotting assay. The results are presented as mean ± SD of three independent experiments (* *p* < 0.05; ** *p* < 0.01).

One previous study demonstrated that *N*-acetyl-l-cysteine, an antioxidant agent, could completely attenuate apoptosis induced by HQ in prostate PC12 cancer cells. The structure of this compound suggests its potential as an antioxidant agent [17]. To examine whether the HQ-induced apoptosis in Molt 4 cells involves the overproduction of ROS, the levels of ROS at different times following HQ treatment were determined. A time-dependent increase in ROS generation was monitored using the carboxy derivative of fluorescein, carboxy-H_2_DCFDA dye. As shown in Figure 6a, HQ treatment (1.25 μg/mL) for 10, 30, 40 and 50 min resulted in 2.67-, 2.87-, 2.27- and 1.05-fold increases in the ROS levels, respectively, as compared with the mean fluorescence index (MFI) of the control. To clarify whether ROS generation is the major regulatory factor in HQ-induced apoptosis, Molt 4 cells were pretreated with 1.5 mM *N*-acetyl-l-cysteine (NAC), an ROS scavenging agent, aiming to suppress the intracellular oxidative stress. The apoptotic population was measured via Annexin V/PI staining after treatment. As shown in Figure 6b, the result of NAC treatment is similar to the negative control group, showing less than 5% of the apoptotic population (data not shown). In addition, the pretreatment with 1.5 mM NAC decreased the apoptotic cell population from 38.63% and 69.6% to 11.45% and 11.33% in response to the use of 0.625 and 1.25 μg/mL of HQ, respectively. These results indicated that blocking the oxidative stress by NAC resulted in saving Molt 4 cells from apoptosis induced by HQ. To further confirm if the MMP disruption induced by HQ is initiated by ROS overproduction, the population of the cells with disturbed MMP in response to HQ treatment with or without NAC pretreatment was determined. The determination of the cell population with disturbed MMP was achieved utilizing a cationic dye, JC-1 (Figure 6c). Cells were divided into four groups, in which two groups were only treated with 0.625 and 1.25 μg/mL of HQ, whereas the other two groups were treated with NAC (1.5 mM) followed by 0.625 or 1.25 μg/mL of HQ. After 24 h, the change in the cell population with disturbed MMP was evaluated. The NAC pretreatment diminished the population of cells with disturbed MMP from 34.3% and 53.02% to 11.9% and 12.4% in response to the treatment with 0.625 and 1.25 μg/mL of HQ, respectively. Furthermore, the effect of NAC pretreatment on the expression of the apoptosis-related proteins was determined. As shown in Figure 6d, NAC pretreatment abrogated PARP cleavage, as well as the activation of caspases 3 and 7. In agreement with the preceding results of Annexin V/PI staining, these findings indicated that the cytotoxic effect of HQ in Molt 4 cells is mediated through apoptotic induction, as well as mitochondrial dysfunction involved in ROS production.

## 3. Discussion

Halenaquinone (HQ), the major active constituents of marine *Petrosia* sp. sponge, exhibited a plethora of promising biological effects, including antifungal, antimalarial, cardiotonic and cytotoxic activities, as well as topoisomerase and protein kinases inhibitory activities [14,15,16,17,18,26]. In previous reports, the cytotoxic activity of HQ was evaluated against A431 (American vulval-derived epidermoid carcinoma), Nakata (Japanese oral squamous cell carcinoma), PC12 (rat pheochromocytoma), KB (human epithelial carcinoma) and L1210 and P388 (mouse lymphocytic leukemia) cells [14,17,21]. In the current report, the cytotoxic activity of HQ against other cancer cell lines was evaluated showing potent activity against leukemia Molt 4 cells.

HQ effectively suppressed the viability and induced apoptosis through mitochondrial dysfunction of the leukemia cells in a dose-dependent manner. Additionally, it was found that HQ is selective towards leukemic cancer cells, because it was less toxic to rat normal lymphocytes, even at the highest tested dose. Furthermore, the xenograft animal results demonstrated that the marine polyketide, HQ, exhibited a potent *in vivo* antileukemic effect, as demonstrated by reducing the tumor weight and size (*p* < 0.05) without any significant effect on mice body weights (Figure 1 and Figure 2). All of the values of the biochemical factors in plasma were within the acceptable range (data not shown).

Previous studies reported that the marine sponge polyketides, halenaquinone and xestoquinone, acted as inhibitors of PI3 kinase, protein tyrosine kinase and topoisomerase [15,17,21,24,26]. In the current study, the inhibitory effect of HQ on HDAC was demonstrated for the first time. The potent inhibitory effect of HQ on topoisomerase and HDAC activity with the IC_50_ values at 0.0055 and 2.905 μg/mL, respectively, suggested its potential to be developed as a cytotoxic agent acting through the inhibition of key enzymes in cancer development.

HQ treatment increased the acetylation of H3K18 and H3 along with the inhibition of HDAC activity (Figure 4a,b). HDAC9, a histone deacetylase responsible for the deacetylation of histone lysine 18 (H3K18), was identified as the target of miR-376a [27]. The miR-376a, which might contribute to cancer development, directly targets matrix extracellular phosphoglycoprotein. The overexpression of miR-376a reduces G2 arrest of the cancer cells and sensitizes the cells to DNA damage-induced killing [28]. The protein p85α (PIK3R1) was identified as a direct and functional target of miR-376a in Huh7 cells. On the other hand, the downregulation of miR-376a may contribute to the development of HCC (hepatoma carcinoma cells) by targeting p85α. As a key regulatory factor of the PI3K pathway, Akt, which has an antagonistic apoptotic function, can translocate from the cytosol to mitochondria to suppress the opening of the permeability transition pore and maintain mitochondrial integrity [29]. Indeed, mitochondrial integrity is a central factor for the cell survival and generation of energy in the form of ATP. Once mitochondrial membrane integrity is breached, mitochondria act as cellular executioners by releasing proapoptotic molecules, leading to the activation of apoptotic cell death [30]. Cancer development is linked to histone hypoacetylation, due to the overexpression of HDACs, and the anticancer activity has been attributed to the restoration of histone acetylation balance [31]. Recently, it was found that vorinostat and entinostat, HDAC inhibitors, exhibited a potent resensitization effect in solid tumors when they were used as components of a combination therapy [32].

Topoisomerases are essential enzymes for cell survival and growth. They even remain tightly bound to chromosomes during mitosis [13]. Recently, poisons of topo IIα, etoposide, doxorubicin and mitoxantrone, have been exploited as the frontline drugs for anticancer treatment in solid tumors and hematological malignancies, but these drugs were found to be implicated in the leukemogenic process [13]. Our results suggested that HQ, the marine natural product, completely inhibited the catalytic reactions of topo I and Iiα, as demonstrated by the cell-free system, and the topo IIα protein that was significantly depleted in HQ-treated Molt 4 cells after 24 h (Figure 2 and Figure 3). A decrease in the related proteins’ expression is reflected in the suppression of the overall catalytic activity [12]. Finally, HQ treatment inhibited the catalytic activity of topo IIα below the threshold levels, inhibiting DNA replication and, consequently, resulting in cell death.

Increasing evidence suggested that mitochondria are the critical source of ROS and could be considered as a potential target in cancer therapy. Many naturally-derived compounds were found to effect mitochondrial activity, such as curcumin, colchicine, paclitaxel and vinca alkaloid-derivative SK228 [33,34,35,36]. Alternations in the mitochondrial functions via the increase of ROS generation and disruption of mitochondrial membrane potential can lead to cell death [35]. In addition, mitochondria are regarded as the crucial organelles in determining cell fate. In this study, HQ treatment initially enhanced the intracellular ROS generation preceding mitochondrial depolarization, which is blocked by NAC pretreatment, a scavenger of ROS (Figure 6a,b). The regulation of the oxidative stress is an important factor in both tumor development and responses to anticancer therapies. However, high ROS levels are harmful to cancer cells, and thus, oxidative stress can result in a tumor-suppressive effect [37]. Recently, lines of evidence indicated that the modulation of oxidative stress could be a valuable anticancer strategy [37]. Importantly, the treatment with HQ increased ROS generation and resulted in mitochondria-dependent apoptosis. The upregulation of proapoptotic proteins (Bax and cytochrome *c*) and downregulation of survival proteins (Akt and Bcl-2) were important consequences of HQ treatment (Figure 5e,f). Consequently, these events activated the caspase cascade and PARP degradation. The association of hexokinase with mitochondria was correlated with the ability of Akt to inhibit apoptosis. It is known that the antiapoptotic protein, Akt, might preserve the integrity of the mitochondria to regulate cellular energy metabolism and survival against stress [38]. Our result suggested that HQ-induced mitochondrial dysfunction involved the inhibition of HK II and p-Akt, leading to apoptosis in the cancer cells.

## 4. Experimental Section

### 4.1. Bioassay Materials

The cell lines were obtained from the American Type Culture Collection (ATCC, Manassas, VA, USA). Cells were maintained in RPMI 1640 medium supplemented with 10% fetal calf serum, 2 mM glutamine and antibiotics (100 units/mL of penicillin and 100 μg/mL of streptomycin) at 37 °C in a humidified atmosphere of 5% CO_2_. RPMI 1640 medium, fetal calf serum (FCS), trypan blue, penicillin G and streptomycin were obtained from GibcoBRL (Gaithersburg, MD, USA). Dimethyl sulfoxide (DMSO), 3-(4,5-dimethylthiazol-2-yl)-2,5-diphenyl-tetrazolium bromide (MTT) and all other chemicals were purchased from Sigma-Aldrich (St. Louis, MO, USA). Antibodies against c-PARP, p-PTEN (Ser^380^), p-GSK 3β (Ser^9^), p-Akt (Ser^473^) and hexokinase I and II were purchased from Cell Signaling Technologies (Beverly, MA, USA). Antibodies for Bax, Bcl-2, Bid, cytochrome *c*, topoisomerase IIα and β-tubulin were obtained from Santa Cruz Biotechnology (Santa Cruz, CA, USA). JC-1 cationic dye and the carboxy derivative of fluorescein (carboxy-H_2_DCFDA) were purchased from Molecular Probes and Invitrogen technologies (Carlsbad, CA, USA). Anti-mouse and rabbit IgG peroxidase-conjugated secondary antibody were purchased from Pierce (Rockford, IL, USA). The Annexin V-FITC/PI (propidium iodide) kit was from Strong Biotech Corporation (Taipei, Taiwan). The Hybond ECL transfer membrane and ECL Western blotting detection kits were obtained from Amersham Life Sciences (Amersham, UK).

### 4.2. Preparation of Halenaquinone Stock Solution

Halenaquinone was isolated and purified from the marine sponge, *Petrosia* sp., and its chemical structure was identified by the interpretation of its spectral data (^1^H-NMR, ^13^C-NMR and 2D NMR), and compared to the previous report [18]. This compound was dissolved in DMSO at a concentration of 10 μg/μL and diluted before use.

### 4.3. MTT Proliferation Assay

Cells were seeded at 4 × 10^4^ per well in 96-well culture plates before treatment with different concentrations of the tested compound [22]. After treatment for 24, 48 or 72 h, the cytotoxicity of the tested compound was determined using the MTT cell proliferation assay (thiazolyl blue tetrazolium bromide, Sigma-M2128, St. Louis, MO, USA). Light absorbance values (OD = OD_570_ − OD_620_) were recorded at 570 and 620 nm using an ELISA reader (Anthoslabtec Instrument, Salzburg, Austria) for calculating the concentration that caused 50% inhibition (IC_50_), *i.e.*, the cell concentration at which the light absorbance value of the experimental group is half that of the control group. These results were expressed as a percentage of the control ± SD established from *n* = 4 wells per experiment from three independent experiments.

### 4.4. Annexin V/PI Apoptotic Assay

The externalization of phosphatidylserine (PS) and membrane integrity were quantified using an Annexin V-FITC staining kit (Strong Biotech Corporation, Taipei, Taiwan) [22]. In brief, 10^6^ cells were grown in 35-mm diameter plates and were labeled with Annexin V-FITC (10 μg/mL) and PI (20 μg/mL) prior to harvesting. After labeling, all plates were washed with a binding buffer and harvested. Cells were resuspended in the binding buffer at a concentration of 2 × 10^5^ cells/mL before assessment on a FACS-Caliburflow cytometer (Beckman Coulter, Taipei, Taiwan) and analyzed with CellQuest software. Approximately 10,000 cells were counted for each determination.

### 4.5. Determination of ROS Generation and MMP Disruption

These assays were performed as described previously [22]. MMP disruption and ROS generation were detected with JC-1 cationic dye (5 μg/mL) and the carboxy derivative of fluorescein (carboxy-H_2_DCFDA, 1.0 mM), respectively. In brief, the treated cells were labeled with a specific fluorescent dye for 30 min. After labeling, cells were washed with PBS and resuspended in PBS at a concentration of 1 × 10^6^ cells/mL before analysis via flow cytometry.

### 4.6. Assay of Topoisomerase I and II Catalytic Inhibitors and Poisons

The assay was performed as described previously [22] and following the manufacturer’s protocol. Standard relaxation reaction mixtures (20 μL) containing 50 mM Tris-HCl (pH 8.0), 10 mM MgCl_2_, 200 mM potassium glutamate, 10 mM dithiothreitol, 50 μg/mL bovine serum albumin, 1 mM ATP, 0.3 μg of pHOT1 plasmid DNA, 6–8 units of human topo I or II (Topogen, Columbus, OH, USA) and the indicated concentrations of etoposide and HQ were incubated at 37 °C for 30 min. Reactions were terminated by adding 2 μL of 10% SDS to facilitate trapping the enzyme in a cleavage complex, followed by the addition of 2.5 μL of proteinase K (50 μg/mL) to digest the bound protein (incubated at 37 °C for 15 min) and, finally, by adding a 0.1 volume of the sample loading dye. The DNA products were analyzed via electrophoresis through vertical 2% agarose gels at 2 voltages/cm in 0.5 × TAE buffer. Gels were stained with ethidium bromide and photographed using an Eagle Eye II system (Stratagene, La Jolla, CA, USA).

### 4.7. Assays of Caspases 8 and 9 Activities

Active caspase activity was determined as previously described [39] and following the manufacturer’s protocol. In brief, after treating the cells with different concentrations of the tested compounds, cells (2 × 10^5^ cells/mL) were collected and washed three times with PBS and resuspended in 50 mM Tris-HCl (pH 7.4), 1 mM EDTA and 10 mM ethylene glycol tetraacetic acid (EGTA). Cell lysates were clarified by centrifugation at 18,000× *g* for three minutes, and clear lysates containing 100 μg protein were incubated with 100 mM of enzyme-specific calorigenic substrates (Ac-IETD-pNA, for caspase 8; Ac-LEHD-pNA, for caspase 9) at 37 °C for 2 h. The alternative activity of caspases 8 and 9 was determined as the cleavage of colorimetric substrate by measuring the absorbance at 405 nm.

### 4.8. Assay of Histone Deacetylase Activity

The assay was performed using the HDAC Inhibitor Drug Screening assay according to the manufacturer’s introductions (BioVision, CA, USA). First, the HDAC colorimetric substrate, which comprises an acetylated lysine side chain, is incubated with different doses of HQ (0.625, 1.25, 2.5, 5 and 10 μg/μL) containing HDAC activity (HeLa nuclear extract) for 30 min at 37 °C. Deacetylation of the substrate sensitizes the substrate, so that, in the second step, the treatment with 10 μL of Lysine Developer produces a chromophore to stop the reaction for 30 min at 37 °C. The fluorescence was recorded at excitation = 350–380 nm and emission= 440–460 nm using a spectrophotometer (Biotec synergy, Vermont, USA).

### 4.9. Human Leukemia Molt 4 Cells Xenograft Animal Model

Establishment of nude mice with xenografts was performed as described previously [40]. Six-week-old male immunodeficient athymic mice were purchased from the National Laboratory Animal and Research Center (Taipei, Taiwan). All of the animals were maintained under standard laboratory conditions (temperature 24–26 °C, 12–12 h dark-light circle) and fed with laboratory diet and water. Molt 4 cells (1 × 10^6^) resuspended in 0.2 mL PBS were injected *s.c.* into the right flank of each mouse, and tumor growth was monitored every day. Fourteen days after tumor cell injection, mice with confirmed tumor growth were randomly divided into two groups. HQ (1 μg/g) was intraperitoneally administered to the treatment group, and the control group received solvent only. HQ was administrated every other day for 31 days. Animals were sacrificed by carbon dioxide. The tumor tissues were carefully dissected from each mouse, and tumor weights were measured. Tumor size was measured three times a week using calipers and tumor volumes were calculated according to the standard formula: width^2^ × length/2.

### 4.10. Generation of Rat Lymphocytes

Lymphocytes were generated from rat PBMCs as described previously [41], with some modifications. In brief, PBMCs were obtained from one whole blood by centrifugation with Ficoll-Hypaque (Amersham Biosciences, Uppsala, Sweden) density gradient centrifugation. The PBMCs were cultured in RPMI 1640 medium containing 10% fetal calf serum, l-glutamine (2 mM), streptomycin (100 μg/mL) and penicillin (100 U/mL) for 2 h. The non-adherent cells were collected, and the T lymphocytes were purified by nylon wool separation, as the source of mononuclear T lymphocytes. Cells were loaded into the column, and the eluted non-adherent cell fraction was rich in T-cells [42].

### 4.11. Statistics

The results were expressed as the mean ± standard deviation (SD). The comparison in each experiment was performed using an unpaired Student’s *t*-test, and a *p*-value of less than 0.05 was considered to be statistically significant.

## 5. Conclusions

In this study, we illustrated the antileukemic activity of HQ, obtained from the EtOAc extract of the marine *Petrosia* sp. sponge. The activity was demonstrated using *in vitro* cellular and *in vivo* animal models. With the cell-free system assay, HQ exhibited potent inhibition of topoisomerase and HDAC activity with IC_50_ values at 0.0055 and 2.905 μg/mL, respectively. Overall, our results showed that HQ treatment led to the inhibition of the tumor growth of leukemia Molt4 cells in the xenograft model, which might be mediated by apoptosis. The results indicated that HQ treatment resulted in ROS overproduction, the suppression of prosurvival proteins expression (PI3K/Akt, Bcl-2 and hexokinase II) and mitochondrial dysfunction.
